# Non-Operative, Micro- and Minimally Invasive Methods for Caries Treatment—A Narrative Review

**DOI:** 10.3390/jcm15041534

**Published:** 2026-02-15

**Authors:** Veselina Todorova

**Affiliations:** Department of Operative Dentistry and Endodontics, Faculty of Dental Medicine, Medical University of Plovdiv, 4000 Plovdiv, Bulgaria; veselina.todorova@mu-plovdiv.bg

**Keywords:** minimally invasive dentistry, dental caries treatment, biomimetic materials

## Abstract

The management of dental caries has evolved from the traditional mechanical approach of “extension for prevention” to a biologically oriented philosophy centered on preserving natural tooth structures. Minimally invasive dentistry (MID) emphasizes early detection, risk assessment, prevention, and conservative intervention based on the lesion’s activity and depth. This review outlines current evidence on non-operative, micro-invasive, and minimally invasive strategies, including fluoride therapy, remineralizing agents such as casein phosphopeptide–amorphous calcium phosphate (CPP-ACP), self-assembling peptides that promote biomimetic enamel repair, sealants, and resin infiltration. Minimally invasive operative methods employ advanced technologies for selective tissue removal—chemomechanical systems (Carisolv, Papacarie, Brix3000), sono-and airabrasion, and new-generation polymeric and ceramic burs (SmartBur, Cerabur) designed to preserve sound dentin. Laser photoablation, particularly with erbium lasers (Er:YAG, Er,Cr:YSGG), enables precise cavity preparation with minimal thermal and mechanical stress. These approaches enhance patient comfort, reduce anesthesia requirements, and maintain tooth vitality. Despite limitations related to cost, equipment, and operator sensitivity, MID represents not only a set of refined clinical techniques but also a comprehensive, evidence-based treatment philosophy founded on biological principles, structural preservation, and the promotion of long-term oral health.

## 1. Introduction

At present, no restorative material has been developed that can fully replicate the structural, functional, and esthetic properties of natural enamel and dentin. Therefore, the maximal preservation of dental tissues remains a fundamental principle in contemporary treatment planning. This concept underpins the philosophy of minimally invasive dentistry (MID), whose core principles include early diagnosis, prevention, and—when necessary—minimal intervention aimed at conserving sound tooth structures [1].

More than a century ago, G.V. Black systematized the prevailing knowledge of the carious process and established the foundational principles of operative dentistry. His seminal four-volume work defined cavity design and restorative protocols that were remarkably comprehensive and scientifically justified for their time, maintaining dominance in clinical practice for decades thereafter. However, the recent years have witnessed substantial scientific and technological progress, leading to an inevitable paradigm shift from Black’s mechanical and extension-based principles toward a biologically oriented, tissue-preserving approach [2].

Dental caries is now recognized as a dynamic, biofilm-mediated process resulting from a disruption in the balance between demineralization and remineralization at the tooth surface. The disease progresses through distinct stages—beginning with reversible, non-cavitated enamel lesions, and advancing to irreversible cavitated lesions if left unmanaged. This progression ultimately results in structural loss and functional compromise of the tooth, affecting esthetics, mastication, phonation, and biological integrity [3].

Advances in understanding the pathogenesis of caries have redefined operative dentistry into a medical and biological discipline, emphasizing early detection, risk assessment, preventive intervention, and conservative management. This evolution reflects a broader shift from surgical repair to disease control and tissue preservation, consistent with the overarching goals of modern minimally invasive dentistry [2,3].

In accordance with the philosophy of minimally invasive dentistry, the management of dental caries is guided by the extent of lesion progression, the degree of tissue involvement, and the potential for remineralization. Treatment strategies are therefore categorized based on their invasiveness and therapeutic objective—ranging from non-operative preventive measures aimed at controlling the biofilm and promoting remineralization, to micro-invasive techniques for sealing or infiltrating early lesions, and minimally invasive operative methods designed to conservatively remove infected tissue while preserving sound dentin and enamel [3].

The objective of this narrative review was to summarize current evidence on non-operative, micro-invasive, and minimally invasive approaches for the management of dental caries and to present a structured classification of contemporary treatment strategies based on their level of invasiveness and clinical indications.

## 2. Materials and Methods

### 2.1. Study Design

This article was designed as a narrative review of the contemporary literature addressing non-operative, micro-invasive, and minimally invasive approaches to the management of dental caries. To enhance transparency and reproducibility of the literature identification and selection process, the review was conducted in accordance with the PRISMA 2020 recommendations, where applicable to narrative reviews; however, the protocol was not prospectively registered (Table S1).

The review was guided by the following research question: What contemporary methods for dental caries management are reported in the literature, and how can they be categorized according to the level of invasiveness?

### 2.2. Literature Search Strategy

A comprehensive electronic literature search was conducted in the PubMed and Scopus databases as primary sources. Google Scholar was used only as a supplementary tool to identify additional relevant publications through citation tracking and manual screening.

The search covered publications from January 2000 to March 2025, reflecting the period of major conceptual and technological developments in minimally invasive dentistry.

The search strategy included combinations of free-text terms and Medical Subject Headings (MeSH) using Boolean operators (AND/OR). Core search terms included “minimally invasive dentistry” OR “minimal intervention dentistry” combined with “dental caries management” OR “caries treatment” and specific intervention-related terms such as “remineralization”, “CPP-ACP”, “self-assembling peptides”, “caries infiltration”, “fissure sealants”, “chemomechanical caries removal”, “air abrasion”, “sonoabrasion”, and “laser-assisted caries treatment”. The search strategy was adapted as appropriate for each database.

Reference lists of relevant articles were also manually screened to identify additional publications of relevance.

### 2.3. Eligibility Criteria

Publications were considered eligible if they:

- addressed the prevention, arrest, or treatment of dental caries using non-operative, micro-invasive, or minimally invasive approaches;
- provided clinically relevant, experimental, or translational data related to caries management;
- included in vitro studies, clinical trials, observational studies, consensus documents, systematic reviews, narrative reviews, or authoritative textbooks;
- were published in peer-reviewed journals or recognized academic sources.

Publications were excluded if they:

- were not related to caries management interventions;
- focused exclusively on conventional invasive restorative techniques without a minimally invasive perspective;
- lacked sufficient methodological or clinical relevance to the scope of the review;
- were unavailable in full text or published in languages inaccessible to the author.

### 2.4. Study Selection Process

**Figure 1:**
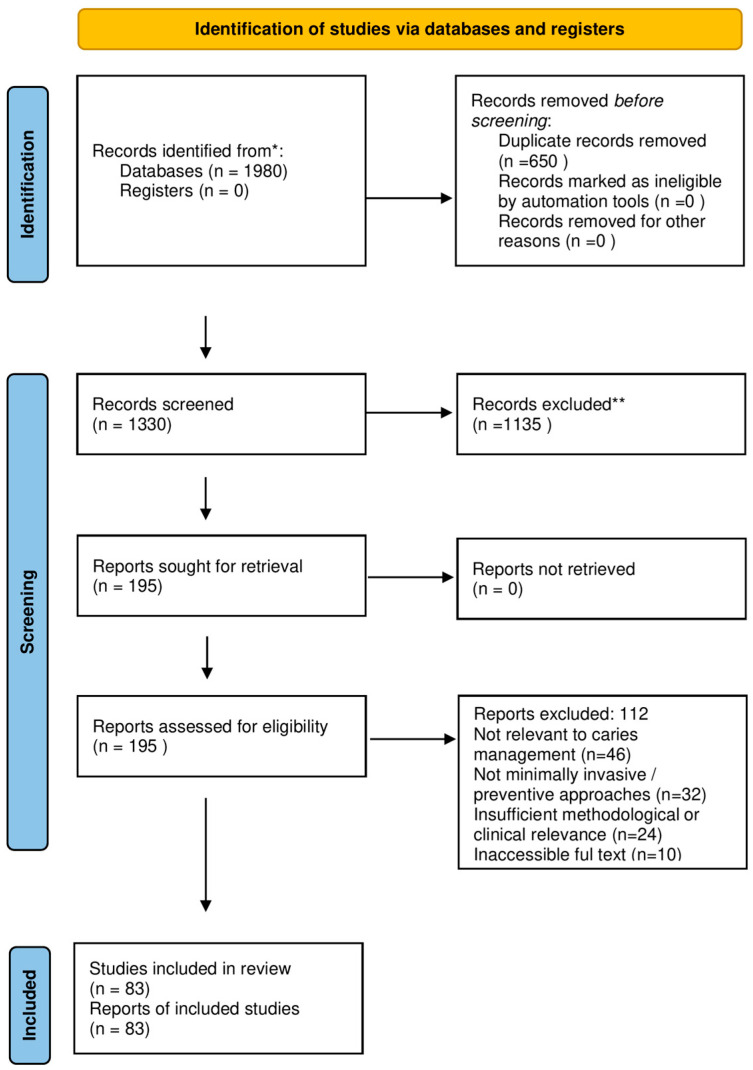
PRISMA Flow diagram of the search, selection, inclusion, and exclusion of articles in the review. * Records identified from electronic databases (PubMed and Scopus) and supplementary searching via Google Scholar (citation tracking/manual screening). ** Records excluded after title/abstract screening.

All records retrieved from the databases were screened by title and abstract for relevance. Potentially eligible publications were subsequently assessed in full text. Duplicate records were removed prior to screening. The study selection process and reasons for exclusion at the full-text stage are presented in the PRISMA 2020 flow diagram (Figure 1).

For the purposes of PRISMA reporting, all peer-reviewed publications that directly informed the conceptual framework, clinical rationale, and discussion of non-operative, micro-invasive, and minimally invasive caries management were considered included studies.

### 2.5. Data Extraction and Synthesis

Data extraction was performed qualitatively by the author. Extracted information included:

- study type and design;
- investigated materials, techniques, or technologies;
- clinical indications and outcomes;
- relevance to minimally invasive caries management.

Due to the heterogeneity of study designs, outcome measures, and methodological approaches, a quantitative synthesis or meta-analysis was not performed. Instead, the findings were synthesized narratively and structured according to the level of invasiveness: non-operative, micro-invasive, and minimally invasive techniques.

### 2.6. Reporting Considerations

As this review did not aim to quantitatively compare interventions or pool effect estimates, formal risk-of-bias assessment and certainty grading were not conducted. This approach is consistent with the narrative nature of the review and the broad scope encompassing experimental, clinical, and conceptual literature.

## 3. Literature Review

The methods for contemporary caries management included in this review are organized according to their level of invasiveness, as presented in Table 1.

For the purposes of this review: Non-operative approaches refer to preventive and therapeutic strategies that control disease activity without removal of tooth structure (biofilm control, remineralization). Micro-invasive approaches involve minimal surface conditioning and sealing or infiltration of early lesions without tissue removal. Minimally invasive operative approaches include selective removal of infected tissue while preserving affected and sound dental structures. These approaches contrast with conventional invasive treatment, which involves extensive mechanical removal of tooth tissue based on traditional cavity design principles.

### 3.1. Non-Operative Methods

#### 3.1.1. Enamel Remineralization

At the earliest stages, caries management at the molecular level begins with the restoration of the balance between demineralization and remineralization. Mineral supply is provided through products containing fluorides and calcium-phosphate compounds, as well as new approaches.

##### Fluorides

Fluoride remains the cornerstone of modern preventive dentistry. It affects the carious process through three main mechanisms [4]: (1) inhibits demineralization; (2) increases enamel resistance and stimulates remineralization by forming fluorapatite; (3) in high concentrations, inhibits bacterial metabolism. It is not yet fully clarified which of these fluoride effects predominates in caries inhibition, considering the dynamic nature of the process. Some authors believe the main action of fluoride is reducing enamel demineralization, while others consider accelerated remineralization to be the key mechanism for caries control [5]. Regardless, the role of fluoride ions in caries prevention and treatment is well-proven and extensively documented in the literature [4,6,7,8]. Fluoride therapy is successfully used to control the carious process, to arrest and reverse early carious lesions, and even to stabilize cavitated lesions.

Fluoride is applied in many forms for both professional and home use—toothpastes, rinses, gels, varnishes, and fluoridated water [9]. Topical fluoride application allows flexibility in administration, dosage control, and most importantly, the possibility of designing an individualized fluoride regimen tailored to the patient’s caries activity and risk [10]. The application of fluoride varnish every three months significantly reduces the progression of proximal carious lesions on molars and premolars [11]. The most pronounced reduction in caries progression is observed among patients with moderate caries risk, whereas patients with high caries activity show no significant improvement [11]. A study evaluating the caries-preventive effect of fluoride varnish applied every six months demonstrated an effectiveness of 69% in high-risk areas, 66% in medium-risk areas, and 20% in low-risk areas [11].

Among topical fluoride delivery methods, varnishes provide prolonged contact with the tooth surface and reduced risk of ingestion, making them particularly suitable for high-risk patients and individuals with limited compliance. Fluoride gels and rinses enable the application of higher fluoride concentrations but require greater patient cooperation and appropriate clinical supervision. Fluoridated toothpastes remain the primary population-level preventive measure, while professional fluoride applications should be individualized according to the patient’s caries risk profile.

Individual response to fluoride therapy varies according to caries risk, salivary factors, and behavioral characteristics, highlighting the importance of personalized preventive protocols.

##### Remineralization—CPP–ACP

The uptake of fluoride is influenced by the concentration of calcium and phosphate ions in saliva and the biofilm. For every two fluoride ions, ten calcium and six phosphate ions are required to form one molecule of fluorapatite—Ca_10_(PO_4_)_6_F_2_ [10]. Therefore, the topical application of calcium and phosphate complexes is used to enhance fluoride-mediated remineralization. Although calcium and phosphate in soluble and insoluble forms have certain limitations, after the year 2000, three Ca–P remineralization systems appeared on the market that improved the bioavailability of these ions [5]: (1) casein phosphopeptide–amorphous calcium phosphate (CPP–ACP; Recaldent technology)—a stabilized amorphous calcium phosphate complex; (2) unstabilized amorphous calcium phosphate (ACP; e.g., Enamelon); (3) bioactive glass containing calcium sodium phosphosilicate (NovaMin technology). Since all of these systems are based on calcium–phosphate compounds, their effects mainly depend on increasing the natural remineralizing capacity of saliva and compensating mineral loss.

Recaldent is the most extensively studied system. It was developed by Reynolds at the University of Melbourne [12]. CPP–ACP is a nanocomplex derived from the casein in cow’s milk that binds calcium and phosphate ions in an amorphous form. CPP–ACP exhibits adhesion to both bacterial cell walls and tooth surfaces. During an acid attack, calcium and phosphate ions are released, creating a supersaturated concentration of ions in saliva, which subsequently precipitate as a calcium–phosphate complex on the tooth surfaces. Recaldent (CPP–ACP) is available in several delivery forms, including chewing gum, topical creams (Tooth Mousse/MI Paste), and a fluoride-containing version (MI Paste Plus) (GC Corporation, Tokyo, Japan). Clinical studies on CPP–ACP chewing gum have demonstrated that it is an easy, inexpensive, and effective method for treating early enamel lesions [8]. Another study confirmed the remineralizing effect of CPP–ACP gum, noting that even when it contains sugar, it can still arrest carious lesions [7]. It is recommended that CPP–ACP gum be used four times daily for 20 min or seven times daily for 5 min [12]. One of the best-known commercial preparations containing Recaldent is Tooth Mousse, a dental cream-mousse applied by the patient or professionally by the dentist. Tooth Mousse Plus is Recaldent with incorporated fluoride (0.27%)—CPP–ACPF (casein phosphopeptide–amorphous calcium phosphate fluoride). There are also reports of CPP–ACP incorporation into glass ionomer cements [12]. Available literature on Recaldent indicates that it significantly reduces the progression of coronal caries, enhances remineralization, and can even lead to lesion regression [5,7].

The second remineralization system, Enamelon, has limited data—aside from manufacturer descriptions, the only published information by Reynolds [12] reported its anticariogenic effect in the treatment of root caries.

The third group, NovaMin, has also been less extensively studied compared with other calcium-phosphate–based systems. However, interest in this material has increased in recent years [5], with emerging evidence suggesting not only remineralization potential but also possible antimicrobial effects [4].

There are also agents based on functionalized β-tricalcium phosphate (β-TCP). It represents a low-dose calcium–phosphate system incorporated into a fluoride formulation. This combination creates a protective barrier that prevents premature calcium–fluoride interactions and facilitates targeted delivery of minerals upon application to the tooth surface.

The potential of fluoride and other remineralizing agents to protect the enamel is restricted to the outer 30 µm of the tooth [13]. A true regenerative approach needs to aim at regenerating hydroxyapatite crystals within the subsurface of the carious lesion.

##### Biomimetic Remineralization—Self-Assembling Peptide P11-4

Enamel, a highly mineralized tissue composed of tightly organized hydroxyapatite crystallites arranged in rod-like prisms, is formed once prior to tooth eruption and cannot regenerate naturally thereafter. The concept of biomimetic remineralization has emerged to address this challenge by emulating the natural enamel mineralization process using synthetic or biologically inspired molecules. Various remineralization systems have been developed based on amelogenin, peptides, amino acids, and calcium phosphate nanoparticles. Self-assembling peptides are among the most studied biomimetic remineralization agents. Peptide-based materials represent an especially promising class of compounds due to their relative ease of synthesis, and most importantly, they have the same chemical structure as biological signals.

The scaffold structure facilitates the targeted deposition of calcium and phosphate ions, leading to mineral precipitation and lesion body remineralization [14,15]. This process results in measurable increases in microhardness, indicating successful subsurface repair of the enamel [14,15,16]. The technology’s foundation in biological mimicry underscores its capacity to support de novo mineral regeneration, a significant advancement over traditional topical treatments. The in vitro study by Schmidlin et al. [13] showed enamel rehardening up to the depth of 200 µm after the application of P11-4, indicating crystallization of mineral, which is taken up by the remineralizing surface.

P11-4 is commercially available under the trade names Curodont Repair and Curodont Protect, developed by Credentis (Windisch, Switzerland). The primary distinction between these formulations lies in their composition and intended use. Curodont Repair comprises the monomeric form of the self-assembling peptide. Curodont Protect contains a polymeric matrix enriched with 1000 ppm P11-4, 900 ppm fluoride, and calcium phosphate, and is suitable for both professional and at-home application. According to the manufacturer’s protocol, the clinical application of Curodont Repair involves several steps: (1) isolation—cheek retractors and cotton rolls or rubber dam; (2) cleaning of organic debris with 2% sodium hypochlorite for 20 s to remove the organic pellicle. Rinsing with water; (3) etching with 35% phosphoric acid for 20 s.; rinsing; (4) application of P11-4 and waiting for 3–5 min for diffusion into the lesion. One applicator is used per lesion.

P11-4 has demonstrated an excellent safety profile in both in vitro and in vivo settings. Studies have consistently shown no evidence of cytotoxicity or immune reactions associated with its use [13].

Compared to CCP-ACP, P11-4 treatment resulted in a progressive and uniform rise in the Ca:P ratio over a 3-month period, indicating sustained mineral deposition and hydroxyapatite formation. Across various in vitro comparisons with fluoride-based treatments, P11-4 consistently demonstrated superior remineralization potential [17,18,19,20].

The P11-4-treated carious lesions showed a significantly improved visual appearance and increased radiographic opacity, remaining stable even 6–12 months after treatment [21]. Randomized controlled trials demonstrated that biomineralization facilitated by P11-4 in combination with fluoride is safe and more effective than the present clinical gold standard of fluoride treatment alone. This was attributed to the ability of self-assembling peptide (SAP) to penetrate into the subsurface lesion and build newly formed hydroxyapatite crystals from bottom to top, unlike fluoride, which cannot remineralize beyond the surface zone [13]. The size of early carious lesions treated with P11-4 was significantly reduced, and the result was superior to that of fluoride varnish [22,23]. In cases of post-orthodontic white spot lesions, SAP showed superior remineralization properties when mineral content was measured through radiodensity and digital subtraction radiography, as in the case series by Abdel Aziz Schlee et al. [21]. The authors presented results showing in-depth remineralization of lesions after a 6-month follow-up and up to 1 year from Curodont Repair application. Randomized clinical trials have demonstrated that self-assembling peptide P11-4 can promote lesion regression and improve mineralization in early enamel caries on both smooth and occlusal surfaces [22,23]. An in situ clinical trial, by Jablonski–Momeni et al. [24], assessed mineral content using micro-CT, which showed a significant increase in mineralization when compared with the fluoride control group.

Kamh et al. [25] and Metwally et al. [26] measured early enamel lesion changes visually, via ICDAS II scoring, and SAP showed a statistically significant enhancement in lesion reversal over fluoride. Another clinical study on the remineralizing efficacy of P11-4 on initial carious lesions on smooth surfaces exhibited reduced lesion depth after a period of 6 and 12 months [27]. A retrospective cohort study evaluating bitewing radiograph assessed the effectiveness of P11-4 on noncavitated carious lesions in a practical clinical setting and reported that the proportion of lesions with no cavitation was 0.96 after 1 year and 0.91 after 2 years [28]. Several recent systematic reviews and meta-analyses conclude that both in vitro and in vivo studies on P11-4 prove the remineralizing potential of the protein and show evidence of superior biomimetic remineralization by SAP P11-4 compared to other remineralizing agents [29,30,31].

Although laboratory and short-term clinical studies demonstrate promising remineralization effects, long-term clinical evidence on lesion stability and durability remains limited.

#### 3.1.2. Agents for Disinfection and Sterilization of Carious Lesions

The elimination of microorganisms within a carious lesion, or altering the ecological niche to promote remineralization, helps arrest the pathological process [32]. Two main methods are described:

##### Ozone

Ozone (O_3_) occurs naturally through the photodissociation of molecular oxygen (O_2_) into activated oxygen atoms. The radicals formed are powerful oxidants with bactericidal, virucidal, fungicidal, and anti-inflammatory properties [32]. This rapid effect is clinically important because it eliminates the possibility of microorganisms developing resistance [32]. Studies show that ozone destroys microbial cell walls within seconds—90% of cariogenic flora are eliminated after 10 s, and 99% after 20 s [33]. At the same time, ozone neutralizes acids and shifts the pH to alkaline levels—effects that last 4–12 weeks [33]. Ozone can be applied in several therapeutic forms: gaseous (Heal Ozone, Curozone GmbH, Wiesbaden, Germany; Prozone, W&H Dentalwerk, Bürmoos, Austria; Ozicure O_3_ device, Ozicure GmbH, Munich, Germany), ozonated aqueous solutions (for disinfection and sterilization), ozonated oils, and ozonated water [34].

For the stabilization and treatment of reversible carious lesions, ozone is applied for approximately 40 s per visit, depending on the size and depth of the lesion [34]. Numerous studies investigating the effect of ozone on the carious process have demonstrated its pronounced positive role in lesion arrest and remineralization [4,35]. Ozone therapy represents an alternative, non-invasive method for the management of dental caries—it is painless, safe, and well accepted by patients. As an alternative in the prevention and treatment of reversible and irreversible caries, ozone has the following indications: prophylaxis of deep pits and fissures, treatment of initial reversible lesions, root caries, and reduction in hypersensitivity of prepared tooth surfaces.

For arresting and treating reversible lesions, ozone is applied for 10–60 s per visit, depending on lesion size and depth. The ozone device consists of an ozone generator and a control unit for suction and conversion of residual ozone into harmless oxygen. A silicone-cuffed applicator is placed on the tooth surface, creating a mild vacuum seal. After starting the cycle, ozone is applied for the preset time (approximately 40 s), then suctioned and converted into oxygen. The system is designed to prevent leaks—if the cuff is not sealed properly, it does not activate, ensuring operator safety. After ozone application, additional steps include biofilm reduction and stimulation of remineralization. Freedman described the protocol for using ozone in irreversible caries [36]: (1) accurate diagnosis; (2) minimally invasive cavity preparation; (3) application of HealOzone; (4) application of the HealOzone remineralizing solution (from the HealOzone Remineralizing Patient Kit), containing toothpaste, mouth rinse, and spray enriched with fluoride, calcium, phosphate, zinc, and xylitol; (5) placement of glass ionomer cement as an intermediate remineralizing provisional restoration for about one year, followed by final composite restoration; (6) prescription of HealOzone remineralizing kit for at least four weeks of home use [37].

Numerous studies confirm the positive role of ozone in arresting and remineralizing carious lesions [3,33,37]. It is particularly suitable for the treatment of fissure caries and root caries. Ozone therapy is an alternative, non-invasive, painless, and safe method for the treatment of dental caries, readily accepted by patients.

While antimicrobial and short-term lesion arrest effects have been reported, systematic reviews indicate inconsistent clinical outcomes and insufficient evidence for long-term effectiveness.

##### Photoactivated Disinfection

Photoactivated disinfection (PAD) is a method for disinfecting or sterilizing a given surface (tissues, wounds, cavities) through the topical application of a photosensitizing agent (dye) followed by irradiation with laser light of a wavelength that is absorbed by the agent. The destruction of microorganisms occurs without damaging surrounding tissues. Low-intensity laser energy alone is not lethal to bacteria but serves to photoactivate the dye, which releases reactive oxygen radicals that damage microbial membranes and DNA. PAD does not produce harmful thermal effects on the teeth or soft tissues of the oral cavity. It has been shown that PAD effectively kills bacteria in complex biofilms that are resistant to other antimicrobial agents [38]. Clinically, PAD is applied for disinfection of deep carious lesions, made possible by the ability of visible red light to penetrate dentin.

An example of a PAD system is the SaveDent red diode laser (Denfotex Ltd., Inverkeithing, UK) with a wavelength of 635 nm, using tolonium chloride (toluidine blue) as the photosensitizing dye [38]. Its ability to rapidly and effectively sterilize the floor of deep carious lesions (up to 1 mm) allows a conservative approach to the removal of demineralized, infected dentin.

When non-operative measures are insufficient to control lesion progression, micro-invasive techniques may be indicated to stabilize early lesions.

### 3.2. Micro-Invasive Techniques

The sealing of pits and fissures and resin infiltration of proximal and smooth surfaces are non-operative caries management methods. The only intervention performed is etching and adhesive bonding of a low-viscosity resin, which strengthens the enamel and creates a barrier against microorganisms.

#### 3.2.1. Fissure Sealants

Fissure caries of permanent molars account for 80% of caries in children and adolescents [39]. This has logical explanations—a high caries risk during the first three years after tooth eruption, difficulty in cleaning narrow and deep fissures, limited fluoride protection in the depths of fissures, and frequent presence of enamel islands with low mineralization. Simonsen [39] defined fissure sealants as materials that cover caries-prone pits and fissures, forming a micromechanically bonded protective layer that prevents the access of cariogenic bacteria to nutrient sources. Indications for fissure sealing include: (1) narrow fissures; (2) short time after eruption of molars; (3) individuals at increased caries risk.

Most sealants are resin-based (composite)—light- or self-curing, but glass ionomer sealants (GIC) may also be used. GIC sealants are indicated when complete moisture control cannot be achieved, such as with partially erupted molars in children [40]. An additional advantage is fluoride release and remineralization of initial lesions [41]. Although studies comparing the caries-preventive potential of GIC and composite sealants are contradictory, they consistently show lower retention rates for GIC sealants [39]. The most recent data conclude that resin-based sealants demonstrate better retention and caries prevention than GICs [39]. Epidemiological studies confirm that pit and fissure sealing is an effective strategy for occlusal caries prevention [42].

For optimal protection, the entire fissure system must be completely sealed without voids. After sealant application, there is a significant reduction in cariogenic microorganisms in the fissure system and arrest of dentinal lesions. The question of sealing fissures with active caries (possibly extending into dentin) remains controversial. For many years, the fear of caries progression under sealants limited their widespread use in clinical practice. Numerous authors have studied the effectiveness and retention of sealants [43]. The FDI reviewed scientific literature on fissure sealants and concluded [42]: (1) sealant application on permanent molars in children and adolescents is an effective caries-preventive method; (2) caries reduction ranges from 86% after 1 year, 78.6% after 2 years, to 58.6% after 4 years; (3) sealant retention rates are 74–96.3% after 1 year and 70.6–76.5% after 2.8 years.

Most authors recommend fissure sealing as a safe and effective method for the prevention and arrest of pit and fissure caries in both primary and permanent teeth [39,42].

#### 3.2.2. Resin Infiltration

This method was developed by a research group from Charité University, Berlin, and patented in 2009 as the revolutionary product Icon (DMG, Hamburg, Germany) [44]. Resin infiltration represents a non-operative, microinvasive method, forming a bridge between prevention and minimally invasive restorative dentistry [44,45]. Caries infiltration is an innovative method for treating initial caries without cavity preparation, where etching is the only intervention. The technique involves infiltrating the lesion with a low-viscosity, highly penetrating resin, known as an infiltrant [46].

Unlike fissure sealants, which form a diffusion barrier on the enamel surface, this technique creates a diffusion barrier within the lesion body, stabilizing it mechanically [47,48]. Indications: Conservative treatment of enamel lesions (outer E1 and inner E2 half of enamel) and lesions extending into the outer third of dentin (D1). It is used for proximal and smooth-surface caries. The procedure is quick and simple—the entire process takes about 15 min: (1) clean the tooth surface and isolate with a rubber dam; place wedges for tooth separation; (2) etching with Icon Etch (15% hydrochloric acid) for 2 min; (3) rinse for 30 s and dry; (4) apply Icon Dry (99% ethanol) for 30 s, then dry; (5) apply Icon Infiltrant for 3 min, remove excess, and light-cure for 40 s; (6) repeat application of Icon Infiltrant for 1 min and light-cure for 40 s; (7) polish.

##### Principle of Resin Infiltration

An initial carious lesion is characterized by the formation of a lesion body—the most demineralized zone, with micropores that act as diffusion pathways for acid penetration and mineral dissolution. The surface layer remains relatively intact, forming a pseudo-intact barrier that limits resin penetration into the lesion body [46].

To improve penetration, acid etching is used to remove this surface layer. Paris et al. initially tested phosphoric acid for 30 s and found minimal reduction in the surface layer [49], risking insufficient resin penetration and continued lesion progression. In contrast, etching with 15% hydrochlorid acid for 90–120 s achieved complete removal of the pseudo-intact layer and optimal infiltration [49]. Early attempts used commercial adhesives and sealants, which showed limited penetration due to their viscosity. Thus, specialized infiltrants were developed—light-curing resins optimized for rapid capillary penetration [47]. These infiltrants achieve the primary goal of the method—filling the lesion’s micropores, thereby isolating it from further acid attacks and mechanically stabilizing the fragile demineralized enamel after polymerization [47]. Studies demonstrate that infiltrated lesions progress significantly more slowly than untreated lesions in cariogenic conditions. Additional research has examined penetration depth [50], application time [51], and resin composition [52].

Resin infiltration is also used for the treatment of non-caries white spot lesions [46,53]. After infiltration, lesions acquire an appearance similar to surrounding healthy enamel [53]. The masking effect is based on the change in light scattering within the lesion [53]. In non-infiltrated white spots, micropores are filled with air or fluid, and the difference in refractive indices between these and enamel makes lesions appear white and opaque. After infiltration, micropores are filled with resin, whose refractive index closely matches enamel, rendering lesions visually invisible [54].

Advantages. Resin infiltration is an ultra-conservative treatment approach, fully aligned with the principles of minimally invasive dentistry, offering multiple benefits: (1) preservation of healthy dental structures; (2) painless, anesthesia-free procedure; (3) definitive treatment extending tooth longevity; (4) single 15 min session; (5) early intervention for high-risk or low-compliance patients.

In cases where cavitation has occurred and tissue integrity is compromised, minimally invasive operative techniques are required to selectively remove infected tissue and restore function.

### 3.3. Minimally Invasive Techniques

As already discussed, non-cavitated lesions can be completely remineralized throughout their depth and fully healed [55]. Once cavitated, however, the lesion cannot be restored and requires operative intervention. The main goal is to re-establish the integrity of the hard dental tissues and to create a smooth surface that will accumulate minimal plaque [55].

Operative and restorative procedures, viewed through the philosophy of minimal invasiveness, aim at maximal preservation of natural tooth structures and the use of biomimetic materials. Only irreversibly damaged tissues are removed and replaced with adhesive restorative materials, while the adjacent and underlying demineralized tissues—capable of remineralization and healing—should not be removed [56]. Minimally invasive cavity preparations can be performed through traditional mechanical–rotary methods or alternative techniques such as air abrasion, sonic or ultrasonic instrumentation, and lasers.

#### 3.3.1. Mechanical-Rotary Techniques

The mechanical-rotary method remains the most widely used preparation technique. It employs handpieces and burs, whose design has been refined according to the micro-intervention philosophy. Their working parts are smaller and more delicate, with long, thin necks, providing better visibility and control over the amount of tissue removed [57].

Traditional tungsten carbide or carbon steel burs for low-speed handpieces remain the most time-efficient tools for caries excavation [58]. However, they remove too much sound tissue and often cause discomfort and pain due to cutting of vital dentin, as well as pressure, vibration, and heat generation [58]. To address these problems, several products have been introduced:

##### Fissurotomy Burs (SS White, Dental, Lakewood, NJ, USA)

Fissurotomy is an ultraconservative preparation of fissures, similar to the earlier preventive odontotomy or enameloplasty. The goal is to widen or shallow fissures into a cleanable shape or restore them with an adhesive material. When used diagnostically to assess caries presence, the technique is called a “caries biopsy” [59].

The specialized Fissurotomy burs are carbide burs with a V-shaped cutting head, available in three types: Fissurotomy Original, Fissurotomy Micro NTF, and Fissurotomy Micro STF. Their design allows fast, controlled shaping of fissures into a plaque-resistant V-form that can be cleaned, sealed, or restored if caries is present.

##### Polymer Burs—SmartPrep/SmartBur (SS White, Dental, Lakewood, NJ, USA)

These burs are designed for the selective removal of carious dentin while preserving healthy tissue [59]. Made of a special polymer with a Knoop hardness of 50—higher than carious dentin (0–30) but lower than sound dentin (70–90)—SmartPrep burs remove only infected dentin [58,60]. They dull when contacting harder tissues, making them self-limiting [58,61]. Their straight, non-spiral blades reduce vibration and prevent cutting into dentinal tubules [62], minimizing pain and eliminating the need for anesthesia. They are especially useful for students and less experienced clinicians, as they enhance tactile sensitivity and prevent over-preparation [62].

Main drawbacks include rapid wear when contacting harder materials and incomplete caries removal. Studies show more residual caries with SmartPrep than with conventional tungsten carbide burs [61]. The newer SmartBur version (SS White Dental, Lakewood, NY, USA) shows reduced but still present residual caries, likely due to the limited hardness of the polymer [61]. Future improvements should focus on ensuring complete caries removal.

##### Ceramic Burs—CeraBur (Komet Dental, Lemgo, Germany)

These are round burs made of aluminum-yttria-stabilized zirconia ceramic in various sizes [63]. According to the manufacturer, CeraBurs efficiently remove infected soft dentin, improve tactile feedback, and reduce preparation time.

In vitro studies show no significant difference between CeraBurs and traditional burs in terms of total caries removal and preparation time [64]. However, their non-selective cutting nature can leave areas of residual carious dentin if tactile assessment is not performed [60].

#### 3.3.2. Non-Rotary Mechanical Techniques

##### Manual Excavation

Thylstrup and Fejerskov define the use of hand instruments and low-speed rotary burs as the most appropriate method for caries removal, since it allows the operator to feel the difference between the relatively hard affected dentin and the demineralized infected dentin. This minimizes the unnecessary removal of sound tooth tissue [65].

An in vitro study demonstrated that manual excavation is the best method in terms of both efficacy and effectiveness for caries removal, as determined by the autofluorescent technique [66]. In contrast, rotary techniques remove excessive amounts of healthy tissue, though they are faster. The advantages of manual excavation formed the basis for the development of the so-called Atraumatic Restorative Treatment (ART) technique. This approach was created for developing countries, where dental caries often leads to tooth extraction due to the lack of electricity and dental equipment. The ART method uses a small set of hand instruments for excavation and glass ionomer cement (GIC) for filling the prepared cavities. GIC is chosen as a restorative material when moisture control is inadequate and for the fluoride release, inhibiting caries progression [66,67]. Advantages of ART include: (1) preserves sound tooth structure; (2) eliminates the need for anesthesia; (3) suitable for patients with special needs—such as those in remote areas without access to dental care, children with behavioral difficulties, patients requiring sedation or general anesthesia, those with early childhood caries, and medically compromised or elderly patients unable to visit a dental office; (4) can be performed by trained non-dental personnel [66]; (5) combines prevention (fissure sealing or caries arrest) and restoration (removal and filling of cavitated lesions).

Survival data from the first application site—rural Thailand—showed an 88% success rate at three years, comparable to amalgam restorations placed under the same conditions. There were no statistically significant differences between ART restorations in children and adults, nor between those placed by dentists and dental assistants [66]. Newer GIC formulations developed for stress-bearing areas show even better performance. Recent studies conclude that ART restorations can remain effective after up to 10 years in the oral cavity [67].

##### Air Abrasion

Also known as microabrasion or kinetic cavity preparation, this pseudo-mechanical technique uses a high-velocity stream of purified aluminum oxide (Al_2_O_3_) particles, approximately 27 µm in size, propelled under air pressure [59,68,69]. The particles, directed at the tooth surface, abrade it without generating heat, vibration, or noise. Aluminum oxide is the most commonly used abrasive agent because of its high cutting efficiency, chemical stability, low cost, low water affinity, and neutral color [69]. Typically, systems using 27 µm aluminum oxide particles are used for shallow cavity preparation and stain removal. Besides particle type and size, other factors directly influencing cutting efficiency include particle velocity, angle of impact, and the type of dental tissue involved.

A main drawback of caries excavation by air abrasion is that sound dentin is removed more efficiently than carious dentin. Although smaller 27 µm particles remove more carious dentin than larger ones (50–125 µm), cavities in sound dentin tend to be deeper [58]. Because carious dentin is softer, it absorbs part of the particles’ energy, reducing cutting effectiveness. Researchers have explored other types of abrasive particles to improve selectivity [65]: (1) spherical glass beads of various diameters improved removal of artificially demineralized dentin but still removed some sound tissue; (2) polycarbonate powder removed artificial soft dentin selectively without cutting sound tissues, but lacks clinical trials; (3) mixtures of aluminum oxide and hydroxyapatite (3:1, 3–60 µm) demonstrated similar effectiveness to manual excavation; 4) bioactive glass particles (25–32 µm, Bioactive glass (NovaMin^®^, NovaMin Technology Inc., Alachua, FL, USA))showed reduced risk of removing sound dentin and selective removal of stained or demineralized enamel [70].

Indications include: (1) small cavity preparations; (2) fissure exploration; (3) stain removal; (4) composite or ceramic restoration removal; (5) surface roughening for adhesive bonding.

Studies show that adhesive systems applied to air-abraded enamel and dentin yield better bond strength than conventional bur preparation, though acid etching with phosphoric acid remains necessary [60,71].

Advantages include: (1) minimal removal of sound tissue; (2) suitable for small, adhesive cavity designs; (3) reduced need for anesthesia; (4) increased patient comfort due to absence of vibration, noise, and heat [60].

Limitations: Air abrasion requires rubber dam isolation, high-efficiency suction, and protective eyewear for both clinician and patient [69] to prevent inhalation, tissue injury, or ocular trauma. Magnification devices (loupes, microscopes) cannot be used, as high-speed particles may damage them [69]. The technique lacks tactile feedback for depth control; thus, frequent visual monitoring is essential. It should not be used in deep cavities (risk of pulpal exposure), for amalgam removal (due to mercury vapor), or for amalgam and indirect restoration preparations, since air-abraded cavities have rounded angles [69].

##### Ultrasonic Preparation

In 1950, Nielsen reported the use of ultrasound for cutting dental tissues. The mechanism involved transferring the kinetic energy of water molecules to the tooth surface through an abrasive medium during high-frequency oscillation of the tip. The author observed that the harder the tissue, the easier it was to cut [72]. Currently, ultrasonic systems designed for minimally invasive cavity preparation include ergonomic devices that include autoclave-sterilizable cassettes and sets of semi-rounded metallic tips, each with one smooth inactive surface and one diamond-coated active surface. The tips are attached to a water-cooled ultrasonic unit producing vibrations above 20,000 Hz.

Ultrasonic preparation is particularly suitable for proximal areas, where conventional rotary instruments risk damaging adjacent teeth. The presence of a smooth, inactive surface protects neighboring tooth surfaces. Ultrasonic tips can be applied to all tooth surfaces—occlusal, cervical, and proximal [72]. Ultrasonics are also used for minor enamel preparations and finishing of cavity margins. A limitation is that additional rotary instruments are often required for fine preparation [73].

##### Sonoabrasion (Sonic Preparation)

This method uses high-frequency air-driven sonic scalers (below 6.5 kHz) with water cooling. An example is the SonicSys Micro Unit, invented by Hugo, Unterbrink, and Möseler (KaVo DentalGmbH, Biberach, Germany), based on the Sonicflex 2000L/2000N air-scaler handpiece, which oscillates in the sonic range with an elliptical motion of the tips. The tips are diamond-coated on one side and smooth on the other, and water-cooled. They come in three shapes: elongated half-torpedo, small hemispherical (1.5 mm), and large hemispherical (2.2 mm). Optimal torque is around 2 N, as excessive pressure dampens oscillations and reduces cutting efficiency [72].

Originally developed for finishing and contouring restorations, sonoabrasion is now successfully used for microinvasive cavity preparation. Studies show that caries removal by sonoabrasion takes about the same time as manual excavation but significantly longer than traditional bur techniques [60,72,73]. Some carious dentin often remains. The surface morphology of sonically prepared dentin reveals a very fine smear layer, thinner than that produced by rotary instruments [72].

Another sonic system, KOMET Sonic Line (Komet Dental GmbH, Lemgo, Germany), includes specialized tips for fissures (initial caries detection, minimal invasive preparation), veneers, and crown preparations.

A relatively new system, the Cariex System (KaVo Dental GmbH, Biberach, Germany), includes two sets—one with diamond tips for enamel preparation, and another with tungsten carbide tips for dentin excavation.

#### 3.3.3. Chemomechanical Caries Removal

This method is based on the principle of selective removal of carious dentin without affecting healthy tissues.

##### Sodium Hypochlorite—Based Systems—Carisolv (MediTeam AB, Sävedalen, Sweden)

The first commercially available system was Caridex (National Patent Medical Products, New Brunswick, NY, USA). Due to several drawbacks—ineffective caries removal, technical difficulties during placement in the cavity, short shelf life, and high cost—it did not achieve wide clinical use [58]. A new system for chemomechanical caries removal—Carisolv (MediTeam AB, Sävedalen, Sweden), was patented in 1989. It consists of two components: (1) a red gel containing three amino acids (glutamic acid, leucine, and lysine), NaCl, NaOH, and erythrosine (a red dye); (2) a clear liquid containing 0.5% NaOCl [60]. The chemical effect of the system is mainly due to sodium hypochlorite, a strong oxidizing and chlorinating agent with proteolytic properties that dissolves carious dentin. Because NaOCl is highly reactive, it can also damage sound tissues. To ensure that its proteolytic action targets only denatured proteins, the three amino acids are added—they enhance sodium hypochlorite’s effect on carious dentin and reduce damage to healthy tissues. When the two components are mixed, a chemical reaction occurs between chlorine and the amino groups of the amino acids, forming chlorinated amino acids that selectively cleave denatured collagen fibers in carious dentin without affecting the underlying demineralized but not denatured collagen. The softened, infected dentin is then removed manually using specially designed hand instruments. Unlike ordinary excavators, these instruments are used with a scraping motion, are effective in several directions, and do not easily penetrate deeply. A modified colorless Carisolv gel also exists, containing half the amino acid concentration and twice the sodium hypochlorite concentration. Indications for Carisolv include deep carious lesions, root and cervical caries, pediatric caries, patients with dental phobias, and patients with contraindications for local anesthesia.

The chemical effects of Carisolv on the pulp have been found to be harmless. Its alkaline pH (~11) neutralizes acids, exerts a bactericidal effect on cariogenic flora, and promotes faster deposition of hard tissue. A drawback of the method is the longer working time compared to rotary techniques and the frequent need for supplementary conventional instrumentation to gain access to carious tissue [60].

Research on Carisolv’s ability to remove the smear layer is inconsistent. Some authors report almost complete removal with open dentinal tubules [74], while others claim that Carisolv does not remove the smear layer and no open tubules are seen (although that study was conducted on non-carious dentin). A third group reports partial smear layer removal with some open tubules and residual debris. These discrepancies are likely due to the technique’s sensitivity to the clinician’s individual skill. In all studies, however, the amount of smear layer remaining after Carisolv treatment is less than that after mechanical excavation [74].

##### Enzyme-Based Chemomechanical Excavation

The enzyme-based agents break down only denatured collagen, while preserving healthy tooth structure. The enzymes used include papain-based systems such as Papacarie (F&A Laboratório Farmacêutico Ltda., São Paulo, Brazil), Brix 3000 (Brix Medical Science S.A, Buenos Aires, Argentina), and CarieCare (Uni-Biotech Formulations Pvt. Ltd., Bengaluru, India), as well as enzymatic agents such as pepsin, pronase, and collagenase.

Papain is a natural proteolytic enzyme derived from the papaya plant. It acts only on necrotic or infected tissues, leaving sound dentin intact. Papacarie is a gel formulation containing papain, chloramine, and other components. It softens the infected dentin, allowing easy removal with hand instruments, reducing the need for rotary tools and local anesthesia. Brix3000 is a newer formulation with a higher concentration and improved stability of papain, leading to faster and more effective action compared to earlier products. These materials make caries excavation more comfortable, minimally invasive, and suitable for use in children, anxious patients, and situations where conventional drilling is not ideal. Akindele et al. [75] found that Papacarie effectively removed infected dentin and preserved healthy dentin. The study reported reduced pain and anxiety compared to conventional drilling, with no need for anesthesia in most cases. Maashi et al. [76] demonstrated that Papacarie was as effective as mechanical methods in removing carious tissue, while being more selective and less invasive. Gupta et al. [77] and Santos et al. [78] compared Brix3000 with Papacarie and conventional excavation, concluding that Brix3000 required less time for caries removal due to its high enzymatic activity (10%) and neutral pH. Bastia et al. [79] reported that Brix3000 achieved effective caries removal with minimal damage to sound dentin and lower postoperative sensitivity.

An alternative chemomechanical agent for caries removal is an experimental gel (Biosolv (SFC-V/SFC-VIII, 3M ESPE, Seefeld, Germany)), consisting of pepsin in a phosphoric acid/sodium biphosphate buffer. According to the manufacturer, the phosphoric acid dissolves the inorganic component of the carious dentin, allowing pepsin better access to the organic matrix for selective degradation of denatured collagen [60]. The chemically softened tissues are then removed with a metal or plastic hand instrument. To prevent over-excavation, SFC VIII gel is recommended for use with a plastic instrument whose hardness lies between that of healthy and infected dentin. Highly pigmented, arrested caries is more resistant to pepsin degradation, though this is not considered a major limitation. Studies show that the pepsin gel removes carious dentin as effectively as Carisolv [60]. However, there is still insufficient laboratory and clinical data regarding the overall efficacy of SFC VIII.

Although selective removal of infected dentin has been consistently demonstrated, clinical performance may vary due to longer treatment time and operator-dependent factors.

#### 3.3.4. Photoablation—Laser Preparation

The first clinical use of lasers for caries removal involved the ruby laser, which exhibited weak caries-removal capacity, excessive temperature rise, overheating of the target area, melting of mineral structures, carbonization of the organic matrix, and frequent pulp necrosis. Laser systems use various physical media to generate different wavelengths, each interacting with specific tissue molecules [38]. Tissue removal occurs through water evaporation, creating high internal pressure and subsequent microexplosions. Depending on pulse duration, energy, and frequency, the resulting changes in hard tissues may include roughening, crater formation, cracking, melting, and recrystallization [80]. Cavity preparations created with lasers lack a strict geometric configuration and well-defined margins.

Erbium:Yttrium-Aluminum-Garnet (Er:YAG) and Erbium, Chromium: Yttrium-Scandium-Gallium-Garnet (Er,Cr:YSGG) lasers have gained prominence among laser types due to their selective tissue ablation, bactericidal effects, and ability to create smear layer-free surfaces, which improve bond strength and the durability of restorations [80,81]. Both have similar wavelengths (2.94 μm for Er:YAG and 2.78 μm for Er,Cr:YSGG). Although Er,Cr:YSGG is slightly more absorbed by hydroxyapatite, both are very close to the absorption peak of water in the infrared spectrum, resulting in similar interactions with hard tissues [60]. Research indicates that cavities treated with lasers exhibit reduced microleakage, consequently enhancing the durability of restorations [82]. Laser-assisted caries removal significantly diminishes pain perception, thereby reducing dependence on local anesthesia. This is particularly beneficial for pediatric patients and individuals with specialized healthcare needs [83].

A modern high-frequency laser system is the Er:YAG LiteTouch laser (Light Instruments Ltd., Yokneam Illit, Israel). According to the manufacturer, this laser is unique because its entire mechanism is miniaturized (12 cm × 2.3 cm) compared to other systems weighing 30–60 kg and requiring 1 m optical fibers. The 2940 nm wavelength is strongly absorbed by water. The pulsed source delivers photons into a directed water–air spray, causing microexplosions of water droplets. Together with sapphire tips that evenly focus the energy, this allows microinvasive tissue removal with minimal thermal effect due to water’s high absorption coefficient in hydroxyapatite [74]. SEM analysis of dentin surfaces treated with the Er:YAG laser shows micro-roughness and highly retentive enamel surfaces, open dentinal tubules, and absence of a smear layer [74].

Advantages of laser preparation include [80]: (1) minimally invasive; (2) patient comfort (no vibration or unpleasant noise); (3) reduced need for anesthesia; (4) favorable surface for adhesion; (5) pulpal safety.

Despite favorable surface characteristics reported in laboratory studies, clinical outcomes vary with respect to treatment efficiency, cost-effectiveness, and practical implementation.

Beyond the technical characteristics of contemporary technologies, their clinical and practical implications should also be considered. From a clinical and public health perspective, minimally invasive approaches may improve patient acceptance due to reduced discomfort, shorter recovery time, and decreased need for local anesthesia. However, the cost and availability of specialized equipment, such as laser or ozone systems, may limit their widespread implementation.

## 4. Limitations

Although this review provides a comprehensive overview of contemporary non-operative, micro-invasive, and minimally invasive approaches to caries management, several limitations should be considered. The included studies demonstrate considerable heterogeneity regarding study design, diagnostic methods, outcome measures, and follow-up periods, which restricts direct comparison and quantitative synthesis of results. Moreover, a significant proportion of the available evidence is derived from in vitro studies and short-term clinical trials, limiting conclusions on long-term lesion arrest, restoration longevity, and disease control. Variability in caries risk assessment, operator-dependent techniques, and patient-related factors may further influence reported outcomes and limit generalizability.

## 5. Future Directions

Future research should prioritize well-designed randomized controlled trials with standardized diagnostic criteria and long-term follow-up to evaluate the clinical effectiveness and durability of minimally invasive caries management strategies. Particular attention is needed for emerging and less extensively studied approaches, including self-assembling peptides (P11-4), ozone therapy, photoactivated disinfection, sono-abrasion, air-abrasion with alternative bioactive particles, enzyme-based chemomechanical agents, and laser-assisted caries removal. For these technologies, further evidence is required regarding long-term caries arrest, restoration survival, patient-reported outcomes, and cost-effectiveness.

Additional studies should also clarify the clinical indications and comparative effectiveness of selective excavation techniques, polymer and ceramic burs, and combined therapeutic protocols integrating remineralization with minimally invasive operative methods. The integration of digital diagnostic tools, artificial intelligence, and individualized caries risk assessment models represents another important area for future investigation, with the potential to support personalized decision-making and optimize minimally invasive treatment strategies in routine clinical practice.

## 6. Conclusions

In summary, advances in the understanding of caries etiology, together with rapid technological development and the introduction of bioactive and biomimetic materials, have established a new paradigm in restorative dentistry—the era of minimal intervention.

The present review highlights contemporary concepts and techniques for the conservative management of dental caries, emphasizing the integration of non-operative, micro-invasive, and minimally invasive approaches. The wide range of available strategies provides clinicians with the flexibility to select the most appropriate, evidence-based method tailored to the patient’s individual risk profile, lesion characteristics, and clinical context.

Minimally invasive dentistry is not limited to a set of techniques but constitutes a comprehensive therapeutic philosophy centered on the preservation of natural tooth structure and the maintenance of long-term oral health. Accordingly, the conservation of sound dental tissues remains a central objective in contemporary dental practice.

## Figures and Tables

**Table 1:** Methods for caries treatment.

Category	Subcategory/Method	Details/Examples
I. Non-operative methods	1. Enamel remineralization	Fluoride, remineralizing agents (CPP-ACP), P11-4 (Curodont)
	2. Sterilization and disinfection of lesions	Ozone, photoactivated disinfection
II. Micro- invasive methods	1. Fissure sealants	Protective sealants to prevent caries progression
	2. Resin infiltration	Icon Vestibular/Icon Proximal
III. Minimally invasive methods	1. Mechanically rotational—handpieces and burs	Traditional burs, fissurotomy burs, polymer burs, ceramic burs
	2. Mechanically non-rotational	Hand excavation, air abrasion, ultrasound, sono-abrasion
	3. Chemomechanical methods	Sodium hypochlorite-based, enzyme-based
	4. Photoablation—lasers	Er:YAG; Er,Cr:YSGG

## Data Availability

The author confirms that the data supporting the findings of this study are available within the article. Further inquiries can be directed to the corresponding author.

## Supplementary Materials

The following supporting information can be downloaded at: https://www.mdpi.com/article/10.3390/jcm15041534/s1, Table S1: PRISMA Checklist.

## Abbreviations

The following abbreviations are used in this manuscript:

MID	Minimally invasive dentistry
CPP-ACP	casein phosphopeptide–amorphous calcium phosphate
SAP	Self-assembling peptides
Er:YAG	Erbium: Yttrium-Aluminum-Garnet laser
Er,Cr:YSGG	Erbium, Chromium: Yttrium-Scandium-Gallium-Garnet
Ca–P	calcium–phosphorus
O_3_	ozone
O_2_	oxygen
PAD	photo-activated disinfection
GIC	glass ionomer cement
FDI	Fédération Dentaire Internationale
E1	caries lesion in the outer half of enamel
E2	caries lesion in the inner half of enamel
D1	caries lesion in the outer third of dentin
ART	Atraumatic Restorative Treatment
NaOCl	sodium hypochlorite
NaCl	sodium hypochlorite
NaOH	sodium hydroxide
